# Hypertension prevalence, awareness, treatment, and control, in male South Asian immigrants in the United Arab Emirates: a cross-sectional study

**DOI:** 10.1186/s12872-015-0024-2

**Published:** 2015-05-07

**Authors:** Syed M Shah, Tom Loney, Mohamud Sheek-Hussein, Mohamed El Sadig, Salma Al Dhaheri, Iffat El Barazi, Layla Al Marzouqi, Tar-Ching Aw, Raghib Ali

**Affiliations:** Institute of Public Health, College of Medicine and Health Sciences, United Arab Emirates University, PO Box 17666 Al Ain, United Arab Emirates; Ambulatory Health Services. SEHA, Al Ain, United Arab Emirates; Dubai Health Authority, Dubai, United Arab Emirates

**Keywords:** Blood Pressure, Epidemiology, Ethnic Variations, Hypertension, Non-Communicable Disease, South Asians, Transients And Migrants, United Arab Emirates

## Abstract

**Background:**

South Asian males constitute the largest proportion of the United Arab Emirates (UAE) population. Minimal data is available on the prevalence of hypertension among South Asian immigrants in the UAE. We determined the prevalence, associated factors, awareness, treatment, and control of hypertension among male South Asian immigrants from India, Pakistan and Bangladesh residing in the UAE.

**Methods:**

We recruited a representative sample (n = 1375; 76.4 % participation rate) of South Asian adult (≥18 years) immigrant males, including Indian (n = 433), Pakistani (n = 383) and Bangladeshi (n = 559) nationalities in Al Ain, UAE (January-June 2012). Blood pressure, height, body mass, waist and hip circumference data were obtained using standard protocols. Information related to socio-demographics, lifestyle factors, history of diagnosis and treatment of hypertension was collected through a pilot-tested adapted version of the STEPS instrument, developed by the World Health Organization for the measurement of non-communicable disease risk factors at the country level .

**Results:**

Mean age of participants was 34.0 years (95 % confidence interval (CI): 33.4, 34.5 years) and the overall prevalence of hypertension was 30.5 % (95 % CI 28.0, 32.8). In this study, 62 % of study participants had never had their blood pressure measured. Over three quarters (76 %) of the sample classified as hypertensive were not aware of their condition. Less than half (48.5 %) of the sample that were aware of their hypertension reported using antihypertensive medication and only 8.3 % had their hypertension under control (<140/90 mmHg). Hypertensive participants were more likely to be overweight (adjusted odds ratio (AOR) = 1.43; 95 % CI 1.01, 2.01); obese (AOR = 2.49; 95 % CI: 1.51, 4.10); have central obesity (AOR = 2.01; 95 % CI 1.37, 2.92); have a family history of hypertension (AOR = 1.51; 95 % CI 1.05, 2.17); and were less likely to walk 30 minutes daily (AOR = 1.79; 95 % CI 1.24, 2.60).

**Conclusions:**

The prevalence of hypertension in a representative sample of young male South Asian immigrants living in the UAE was relatively high. However, the awareness, treatment, and control of hypertension within this population were very low. Strategies are urgently needed to improve the awareness and control of hypertension in this large population of migrant workers in the UAE.

## Background

High blood pressure, followed by elevated blood glucose, obesity, and abnormal plasma lipids levels are the major modifiable risk factors for cardiovascular mortality in both developed and developing countries [1]. Globally, approximately 17 million deaths a year are associated with cardiovascular diseases and complications related to hypertension account for over 50 % (≈9.4 million) of these deaths [2]. In adults, an increase of 20 mm Hg in usual systolic blood pressure (SBP) or 10 mmHg in usual diastolic blood pressure (DBP) is associated with more than a twofold increase in stroke death rates and a twofold increase in the mortality rates from ischemic heart disease and other cardiovascular causes [3]. In addition, a significant proportion of adults with hypertension have found to be unaware of their hypertension [4]. Indeed, studies have shown that the morbidity and mortality associated with hypertension can be significantly reduced by increasing awareness, treatment, and control of hypertension [5].

Previous studies have shown that South Asian populations (India, Pakistan, Bangladesh, Sri Lanka, and Nepal) contribute the highest proportion of the burden of cardiovascular diseases compared with any other region globally [6]. In addition, risk factors for myocardial infarction, hypertension and type 2 diabetes are developed at a lower age in South Asians than in other ethnic groups [7, 8]. Moreover, South Asian migrants living in other countries had higher death rates from coronary heart disease at relatively younger ages compared to the local population [9, 10].

The United Arab Emirates (UAE) has experienced significant economic, industrial, and population growth, for the last few years. Expatriates account for 80 percent of the UAE population and about two-thirds of the immigrant population are South Asian migrant workers from India, Pakistan and Bangladesh [11]. The majority of South Asian immigrants are unskilled males with low literacy levels, working in low salary jobs, separated from their families in their home country, and living in large labour camps (shared accommodation). Consequently, many of these male South Asian workers suffer from stress, anxiety and depression [12]. Additionally, studies of migrant populations in economically developed countries showed that exposure to environmental factors and a transition in lifestyle may place immigrants at a higher risk for developing metabolic risk factors (e.g. hypertension) for cardiovascular diseases [13, 14]. These studies provide evidence to support the notion of a *Healthy Migrant Effect* seen in Western countries where upon arrival, immigrants are healthier than the native population; however, their health status declines as the duration of their stay increases. Currently, there is a lack of information on the status of risk factors for cardiovascular diseases including hypertension in the South Asian population living in the UAE. This study aimed to determine the prevalence of hypertension, its correlates and the level of awareness, treatment, and control of hypertension among male immigrants from India, Pakistan and Bangladesh residing in the UAE.

## Methods

### Study Design

This study employed a cross-sectional design. Ethical approval was obtained from both the Al Ain Medical District Human Research Ethics Committee, and the Abu Dhabi Ambulatory Healthcare Services Research Committee. Informed written consent was taken from participants.

### Setting

All expatriate workers seeking employment in the UAE are required by federal law to undergo a communicable disease screening test (i.e. tuberculosis by chest X-ray and human immunodeficiency virus by serology), at a government visa screening centre before receiving a residency visa. Expatriates are required to undergo a repeat screening test to renew their visa after three years. Study participants were recruited from the only Disease Prevention and Screening Centre in the city of Al Ain (eastern region of Abu Dhabi, UAE) between January and June 2012.

### Participants and Study Size

In the year 2012, a total of 181231 male immigrants visited the Disease Prevention and Screening Centre (DPSC) in the city of Al Ain to obtain or renew a visa [15]. The sample size of this study was based on the need to explore differences among subgroups. A difference of 1/3 standard deviation in continuous measures such as blood pressure will usually include all levels of public health interest. For a significance level of 5 % and a power of 80 % we would need 144 per group in a two-equal group comparison. We estimated a sample size of 1800 to be adequate to address the primary research question and the majority of the other research questions. We did not include females as they constitute less than 15 % of South Asian immigrants who visit the DPSC to obtain or renew an existing residency visa. Our sampling frame included a list of male expatriate workers from India, Pakistan and Bangladesh who enrolled for medical examination at the only DPSC visa screening centre in Al Ain. In this study we invited every third migrant worker from India, Pakistan and Bangladesh who visited the DPSC in Al Ain for the screening examination to obtain a new or renew an existing visa between January and June 2012 to participate in the study. Of the 1800 eligible participants, 1375 (76.4 %) participated in the study. A substantial number (23.6 %) of eligible migrant workers did not participate due to time constraints as their number was called to complete other visa related medical tests.

### Measurements and Definitions

The study consent forms and questionnaires were written in both Urdu and Bengali. The questionnaire was initially developed in English, and then forward translated to Urdu and Bengali. The questionnaire was then pretested in a pilot study and finalized after necessary amendments. The questionnaire comprised an adapted version of the “STEPS Methodology”, developed by the World Health Organization (WHO) for the measurement and surveillance of non-communicable disease (NCD) risk factors at the country level [16]. Due to the low literacy rates among the South Asian expatriate population in the UAE, all questionnaires were conducted in Urdu or Bengali and interview-led by a native Urdu or Bengali speaking research assistant trained to administer the questionnaire. The questionnaire interview collected information including demographic characteristics, modifiable lifestyle risk factors including tobacco use, alcohol consumption, physical activity, family and personal disease history, home country residence setting (rural, urban, semi-urban), occupation, monthly salary in UAE dirhams (AED; AED 1.00 ≈ USD 3.65) , years lived in the UAE, current type of accommodation, history of current and past cigarette smoking and tobacco chewing, and history of exposure to second-hand tobacco. We classified monthly earning into bottom quartile (AED <900 per month), second quartile (AED 900 to <1200), third quartile (AED 1200 to 2000), and top quartile (AED >2000).

Subjects were classified as current smokers if they answered yes to the question, *“do you smoke cigarettes daily”.* Former smokers were defined as having smoked at least 100 cigarettes in their lifetime. Ever smokers included current and former smokers. The variable relating to alcohol was based on the question, *“did you drink any alcohol during the last 12 months?”* The variables used for the analysis were smoking, use of smokeless tobacco, and alcohol consumption during the last year. Information on physical activity was obtained using the International Physical Activity Questionnaire (IPAQ-short version) [17].

All the research staff members successfully completed a training program that familiarized them with the study objectives, design and methodology. We followed the 1999 World Health Organization/International Society of Hypertension guidelines to measure blood pressure (BP) [18]. Resting brachial BP was measured using a calibrated automated device (Omron HEM-705cp) in sitting position using the right upper arm and an appropriate sized cuff after a period of five minutes rest. The average of the two measures taken was used for analyses. Hypertension was defined as a mean systolic ≥ BP 140, a mean diastolic BP ≥90 or current hypertension treatment with prescription medication (an affirmative response to the question to the following sequence of questions: “Has a doctor ever told you that you had high blood pressure?”; “Because of your high blood pressure, have ever been told to take prescribed medicine?”; and “Are you now taking prescribed medicine for the last two days?” Awareness of hypertension was defined by participant self-report of any previous hypertension diagnosis by a health care professional. Treatment of hypertension was defined as taking antihypertensive drugs within the last two days prior to the interview. Hypertension was considered as controlled among those on treatment if the average BP was ≤140/90 mmHg.

Body mass was measured to the nearest 0.1 kg using a calibrated electronic scale with a mounted stadiometer that was used to measure height to the nearest 0.1 cm (SECA Hamburg, Germany). Body mass and height measurements were completed with the participant wearing light clothing without shoes and standing motionless. Waist and hip circumference were measured using a flexible, non-stretch nylon tape measure (SECA Hamburg, Germany) while subjects were lightly clothed. Waist circumference was measured midway between the lower rib margin and the top of the iliac crest, in centimeters (cm) to the nearest 0.1 cm. Hip circumference was measured at the point of maximal protrusion of the gluteal muscles to the nearest 0.1 cm. Body mass index (BMI) was calculated as body mass in kilograms divided by height in meters squared. World Health Organization cut-offs were used to classify overweight (25.0-29.9 kg/m [2]) and obese (≥30.0 kg/m [2]) adults [19]. We used a waist-to-hip ratio (WHR; waist in cm/hip circumference in cm) ≥0.90 to define central obesity in our male population [20]. The WHO world reference population was used in calculating age-standardized hypertension prevalence rates [21]. In addition, we collected a non-fasting venous blood sample to measure glycated hemoglobin (HbA1c) levels in a random sub-sample (n = 99) of the study population. The WHO cut-off of HbA1c ≥ 6.5 % was used to indicate the presence of diabetes mellitus in the sub-sample.

### Statistical Analysis

Data were entered on a computer with the Microsoft Access software and then imported into Stata version 11.0 (StataCorp LP, College Station, TX) for analysis. Percentages, means and their 95 % confidence intervals were calculated for descriptive purposes. Chi-squared test were conducted for categorical variables and analyses of variance were conducted for continuous variables. Stepwise logistic regression analysis was used to examine the association of socio-demographic, lifestyle and other factors with the odds of hypertension and hypertension awareness. Due to the small sample size we could not conduct multivariable analysis for treatment and control. Two-tailed p-values ≤ 0.05 were considered statistically significant. We included monthly income, education, occupation, residence, smoking and alcohol status as potential confounding variables in our final multivariable model (Table 3).

## Results

Out of 1800 eligible males, 1375 (76.4 %) participated, from India (n = 433), Pakistan (n = 383) and Bangladesh (n = 559). The mean age of study population was 34.0 years (95 % confidence interval (CI): 33.4, 34.5). Table 1a and b show the general characteristics of the study population. Compared to Indian and Pakistani immigrant workers, Bangladeshis were younger and a higher proportion (60.4 %) earned less than AED 1000 per month compared to Indians (20.6 %) and Pakistanis (32.3 %). Bangladeshis and Pakistanis were less educated than Indians (only 13.5 % had college or university level education compared to 31.7 %, respectively). Over two-thirds of the study participants (68.0 %, 95 % CI 66.1, 71.1) were from a rural/village background in their home countries and the majority (70.0 %) of the immigrants were married with an average of three children. More than half (52.2 %) shared accommodation with non-relatives and the remaining participants reported either residing with their sponsor (13.4 %), in single accommodation (11.1 %), in labor camps (12.2 %) or with family members (11.1 %). The majority (55.1 %) of participants had been living in UAE for more than six years. The most common occupational categories of the immigrants included driver (23.1 %), laborer (17.1 %), agriculture worker (17.1 %), construction worker (12.5 %) and sales man (5.7 %).

**Table 1:** Characteristics of the South Asian Immigrants in Abu Dhabi, UAE 2012

Characteristics	India (n = 433)	Pakistan (n = 383)	Bangladesh (n = 559)	p
a				
Age-years (mean)	36.3 (35.3, 37.2)	34.8 (33.7, 35.9)	31.7 (31.0, 32.5)	0.000
Age group-years (%)				
18-35 (years)	50.0 (48.7, 51.3)	59.6 (54.4, 64.7)	71.1 (66.3, 75.7)	0.000
36-45 (years)	28.1 (23.5, 32.7)	20.5 (16.4, 24.9)	20.8 (16.7, 25.1)	
46+ (years)	21.9 (17.8, 26.3)	19.9 (15.8, 24.2)	8.1 (5.1, 11.1)	
Education (%)				
None	3.1 (1.6, 5.0)	23.9 (19.6, 28.4)	12.9 (10.2, 15.9)	0.000
Primary or middle	23.3 (19.2, 27.6)	31.4 (26.7, 36.2)	46.0 (41.7, 50.2)	
Secondary or high school	36.9 (32.2, 41.7)	28.3 (23.7, 33.1)	27.6 (23.8, 31.5)	
College or university	36.7 (31.7, 41.5)	16.4 (12.7, 20.4)	13.5 (6.6, 22.3)	
Income – AED (%)				
Bottom quartile	13.5 (10.4, 16.8)	17.2 (7.7, 21.2)	44.2 (13.6, 48.3)	0.000
Second quartile	19.8 (16.2, 23.7)	21.1 (8.6, 25.4)	22.0 (8.9, 25.5)	
Third quartile	39.5 (34.9, 44.1)	28.2 (10.2, 32.8)	21.5 (8.8, 25.0)	
Top quartile	27.2 (23.2, 31.5)	33.4 (11.4, 38.2)	12.3 (6.4, 15.2)	
Immigrated from a rural village (%)	64.3 (59.3, 69.1)	56.7 (51.3, 61.9)	83.2 (79.7, 86.3)	
Married (%)	78.5 (74.1, 82.5)	75.3 (70.5, 79.7)	60.1 (55.7, 64.3)	
Do not live with immediate family (%)	88.6 (85.0, 91.6)	88.4 (84.7, 91.6)	81.0 (77.4, 84.3)	
Type of accommodation (%)				
Shared with non-relatives	51.4 (46.4, 56.3)	41.1 (36.1, 46.2)	58.6 (54.5, 62.8)	0.000
Shared with family	10.5 (7.6, 13.7)	15.0 (11.8, 19.3)	7.8 (5.6, 10.2)	
Single accommodation	13.3 (10.1, 16.8)	11.1 (8.1, 14.5)	8.9 (6.6, 11.5)	
Live with sponsor	13.3 (10.1, 16.8)	16.7 (13.0, 20.7)	12.2 (9.5, 15.1)	
Live in a labor camp	11.5 (8.5, 14.8)	16.1 (12.5, 20.1)	12.4 (9.7, 15.3)	
Years in UAE (%)				
Up to 1 year	11.2 (8.1, 14.6)	10.5 (7.4, 14.1)	10.3 (7.6, 13.3)	0.000
2 to 5 years	31.3 (26.6, 36.2)	30.5 (25.5, 35.7)	38.6 (34.1, 43.2)	
6 to 10 years	21.2 (17.1, 25.6)	17.1 (13.1, 21.4)	23.1 (19.3, 27.1)	
>10 years	36.3 (31.4, 41.3)	41.9 (36.5, 47.4)	28.0 (23.9, 32.2)	
b				
Occupation (%)				
Driver	21.5 (17.5, 25.7)	33.9 (29.1, 38.8)	19.2 (16.0, 22.7)	0.000
Laborer	14.1 (10.8, 17.2)	12.8 (9.5,16.4)	20.3 (17.0, 23.8)	
Construction worker	12.6 (9.5, 16.1)	8.3 (5.7, 11.4)	13.1 (10.4, 16.1)	
Agriculture worker	8.2 (5.6,11.1)	25.0 (20.7, 29.6)	20.5 (17.2, 24.1)	
Salesman	12.6 (9.5, 16.1)	3.1 (1.6, 5.1)	3.3 (2.2, 5.4)	
Professional, office worker	10.8 (7.9, 14.1)	6.7 (4.4, 9.5)	3.6 (2.2, 5.4)	
Business shop keeper	5.4 (3.4, 7.8)	4.2 (2.4, 6.5)	4.0 (2.5, 5.8)	
Hospitality worker (cook, waiter)	8.2 (5.7, 11.1)	0.8 (0.5, 11.0)	6.5 (4.6, 8.8)	
Tailor	3.6 (1.9, 5.7)	1.9 (0.7, 3.5)	6.8 (4.8, 9.1)	
Other	3.1 (1.6, 5.0)	3.3 (1.7, 5.4)	2.7 (1.5, 4.3)	
Family history of hypertension (%)	14.8 (11.4, 18.8)	15.0 (11.5, 19.1)	23.2 (19.6, 27.1)	0.001
Family history of diabetes (%)	15.9 (12.4, 19.9)	8.1 (5.5, 11.4)	8.9 (6.6. 11.7)	0.000
Body mass index – kg/m [2] (mean)	31.5 (21.1, 41.9)	26.6 (25.1, 27.9)	26.2 (24.2, 28.2)	0.000
Waist-to-hip ratio (mean)	0.93 (0.92, 0.94)	0.93 (0.92, 0.94)	0.92 (0.90, 0.93)	0.033
Waist circumference – cm (mean)	89.5 (88.5, 90.5)	92.9 (90.8, 93.5)	85.6 (84.7, 86.6)	0.000
Smoking (%)	34.3 (29.6, 39.3)	35.8 (30.1, 41.0)	49.1 (44.7, 53.4)	0.000
Smokeless tobacco (%)	5.2 (3.1, 7.9)	11.1 (8.0, 14.9)	15.6 (12.6, 19.1)	0.000
Exposure to second hand tobacco (%)	40.7 (35.8, 45.8)	49.7 (44.4, 55.0)	46.7 (42.4, 51.1)	0.000
Drinking alcohol – ever (%)	20.1 (16.1, 24.4)	3.1 (1.6, 5.5)	7.8 (5.6, 10.4)	0.000
Walk 7 days a week (%)	81.1 (76.7, 84.9)	85.9 (81.9, 89.4)	77.2 (73.3, 80.8)	0.006
Reported vigorous physical activity (%)	17.1 (13.5, 20.6)	18.8 (14.8, 22.7)	18.6 (15.4, 21.8)	0.774
Reported moderate physical activity (%)	20.5 (16.7, 24.4)	18.9 (14.8, 22.7)	37.0 (33.1, 41.0)	0.000
BP measurement – never (%)	57.3 (51.9, 62.4)	62.7 (57.2, 67.9)	68.8 (64.0, 73.2)	0.004
Hypertension (%)	34.5 (30.1, 39.1)	28.2 (23.6, 32.7)	28.8 (25.0, 32.6)	0.073
Physician diagnosed diabetes (%)	10.8 (7.9, 13.8)	6.5 (4.0, 9.0)	6.8 (4.7, 8.9)	0.030
HbA1c ≥ 6.5 %	11.6 (1.6, 21.6)	4.3 (−0.4, 13.4)	9.1 (1.2, 19.4)	0.618
Systolic Blood Pressure – mmHg (mean)	132.6 (130.9, 134.4)	129.2 (127.4, 131.0)	129.1 (127.7, 130.5)	0.003
Diastolic Blood Pressure – mmHg (mean)	79.6 (78.4, 80.8)	75.9 (74.7, 77.2)	76.7 (75.6, 77.7)	0.000

On average, Indians had a higher BMI (31.5 kg/m [2]) compared to Pakistanis (26.6 kg/m [2]) and Bangladeshis (26.2 kg/m [2]). Bangladeshis reported a higher rate of smoking (49.1 %) compared to their Indian (34.3 %) and Pakistani (35.8 %) counterparts. In this study, over half of participants (61.6 % 95 % CI 58.8, 64.4 %), had never had their blood pressure measured and nearly a third of all South Asian males had hypertension (n = 419; 30.5 %; 95 % CI 28.0, 32.9). The overall age-standardized prevalence of hypertension was 34.2 % (95 % CI 31.7, 36.5). The crude prevalence of hypertension varied by the nationality, 34.6 % (95 % CI 30.2, 39.3) in Indians, 28.2 % (95 % CI 23.7, 32.9) in Pakistanis, and 28.8 % (95 % CI 25.1, 32.7) in Bangladeshi immigrants. The age-standardized prevalence of hypertension was 35.7 % (95 % CI 31.2, 40.3) in Indians, 31.3 % (95 % CI 24.8, 38.2) in Pakistanis, and 33.9 % (30.0, 37.8) in Bangladeshi participants.

Only a quarter of participants (n = 99; 23.6 %) classified as hypertensive were aware of their condition and the prevalence of hypertension awareness varied by nationality. The prevalence of awareness was 22.0 % (95 % CI 15.6, 29.4) among Indians, 28.3 % (95 % CI 19.9, 37.8) among Pakistanis and 23.1 % (95 % CI 16.7, 30.4) among Bangladeshis. Of those aware of hypertension, 48 (48.5 %) reported use of antihypertensive drugs in the past two days and only 4 (8.3 %) had their hypertension under control (<140/90 mmHg).

Table 2 show the prevalence of hypertension and awareness and the factors correlated with hypertension prevalence and awareness. The prevalence of hypertension was higher in Indians compared to Pakistani and Bangladeshi immigrants. Hypertension prevalence was lower among those with no formal schooling compared to their literate counters parts. Prevalence of hypertension also varied by occupational category and the lowest prevalence (23.3 %) was noted among agricultural workers and the highest prevalence (40.0 %) among those who were shop keepers or salesmen. The prevalence was lower in people who came from rural villages and was higher among those who had been in UAE for more than 10 years. The prevalence was higher among those who were married, overweight or obese, or had central obesity or did not walk for at least 30 minutes a day. Similarly, hypertensive participants were more likely to be aware of their condition if their age was greater than 35 years, had a higher monthly salary, worked as a hospitality worker, had been in UAE for more than five years, were married, overweight and obese, or had a central obesity.

**Table 2:** Prevalence and crude odds ratios (COR) of factors associated with hypertension prevalence and awareness, in South Asian Immigrants, Al Ain, Abu Dhabi, UAE, 2012 (n = 1,375)

		Hypertension			Awareness		
Characteristics	All	n (%)	COR (95 % CI)	P	n (%)	COR (95 % CI)	P
Nationality							
India	433	150 (34.6)	1.0		33 (22.0)	1.0	
Pakistan	383	108 (28.2)	0.74 (0.55, 0.99)	0.049	30 (28.3)	1.35 (0.76, 2.39)	0.250
Bangladesh	559	161 (28.8)	0.76 (0.58, 0.99)	0.049	36 (23.1)	1.02 (0.60, 1.76)	0.822
Age group							
18-35 years	810	179 (22.1)	1.0		19 (10.9)	1.0	1.000
36-45 years	307	115 (37.5)	2.11 (1.58, 2.81)	0.000	26 (23.2)	2.45 (1.28, 4.68)	0.007
46+ years	200	111 (55.5)	4.39 (3.18, 6.08)	0.000	51 (46.8)	7.13 (3.88, 13.08)	0.000
Monthly income							
Bottom quartile	371	94 (25.3)	1.0		13 (13.9)	1.0	
Second quartile	290	89 (30.7)	1.30 (0.92, 1.83)	0.13	21 (12.1)	1.96 (0.91, 4.210	0.085
Third quartile	399	132 (33.1)	1.46 (1.06, 1.99)	0.02	33 (25.8)	2.13 (1.05, 4.33)	0.035
Top quartile	315	104 (33.0)	1.45 (1.04, 2.02)	0.03	32 (30.8)	2.73 (1.33, 5.61)	0.006
Education							
No formal schooling	173	35 (20.2)	0.61 (0.36, 1.01	0.06	5 (14.3)	0.68 (0.20, 2.33)	0.548
Primary or middle	473	149 (31.5)	1.09 (0.72, 1.66)	0.653	40 (27.4)	1.15 (0.66, 3.65)	0.310
Secondary or high school	586	192 (32.7)	1.16 (0.77, 1.74)	0.459	46 (24.5)	1.33 (0.57, 3.09	0.499
College or university	139	41 (29.5)	1.0		8 (19.5)	1.0	
Occupation							
Driver	317	94 (29.6)	1.38 (0.94, 2.04)	0.097	29 (30.8)	2.97 (1.30, 6.79)	0.010
Laborer	234	71 (30.3)	1.43 (0.95, 2.16)	0.086	9 (13.0)	1.0	
Construction worker	172	64 (37.2)	1.95 (1.26, 3.00	0.002	11 (17.7)	1.43 (0.55, 3.74)	0.457
Agriculture worker	236	55 (23.3)	1.0	1.0	11 (21.1)	1.79 (0.68, 4.70)	0.238
Salesman	79	26 (32.9)	1.61 (0.92, 2.82)	0.092	9 (34.6)	3.52 (1.21, 10.28)	0.021
Professional, office worker	95	29 (30.5)	1.44 (0.85, 2.45)	0.173	7 (23.3)	2.02 (0.67, 6.08)	0.207
Business/shop keeper	60	24 (40.0)	2.19 (1.21, 3.99)	0.010	7 (29.2)	2.74 (0.89, 8.45)	0.079
Hospitality worker	71	19 (26.8)	1.20 (0.65, 2.20)	0.551	8 (42.1)	4.85 (1.54, 15.29)	0.007
Tailor	70	24 (34.3)	1.72 (0.96, 3.06)	0.067	4 (16.7)	1.33 (0.37, 4.80)	0.660
Other	40	12 (30.0)	1.41 (0.67, 2.96)	0.363	4 (36.4)	3.80 (0.92, 15.67)	0.064
Type of accommodation							
Shared with non-relatives	717	232 (32.4)	1.75 (1.17, 2.62)	0.006	55 (23.9)	1.52 (0.59, 3.84)	0.378
Shared with family	152	44 (28.9)	1.49 (0.89, 2.48)	0.122	14 (31.8)	2.25 (0.76, 6.67)	0.141
Single accommodation	153	60 (39.2)	2.36 (1.44, 3.86)	0.001	16 (28.1)	1.89 (0.66, 5.40)	0.237
Live with sponsor	184	46 (25.0)	1.22 (0.74, 2.01)	0.429	8 (17.8)	1.04 (0.32, 3.35)	0.941
Live in a labor camp	168	36 (21.3)	1.0		6 (17.1)	1.0	
Home country setting							
Rural	932	269 (28.9)	1.0		59 (22.3)	1.0	
Urban or semi-urban	426	149 (34.9)	1.32 (1.01, 1.69)	0.02	40 (27.4)	1.32 (0.82, 2.09)	0.245
Length of stay in UAE							
1 to 5 years	547	137 (25.1)	1.0		9 (6.7)	1.0	
6 to 10 years	257	78 (30.8)	1.32 (0.94, 1.81)	0.11	19 (25.3)	4.75 (2.02, 11.15)	0.000
>10 years	414	178 (43.0)	2.26 (1.71, 2.96)	0.000	71 (40.1)	9.38 (4.47, 19.65)	0.000
Marital status							
Single	412	83 (20.1)	1.0		5 (6.3)	1.0	
Married	963	336 (34.9)	2.12 (1.61, 2.79)	0.000	94 (28.2)	1.82 (2.28, 14.85)	0.000
Body mass index – kg/m [2]							
<25.0	758	170 (22.4)	1.0		28 (16.8)	1.0	
25.0 - 29.9	486	182 (37.4)	2.07 (1.61, 2.66)	0.000	46 (25.7)	1.72 (1.01, 2.91)	0.044
≥30.0	129	66 (51.2)	3.62 (2.46, 5.32)	0.000	24 (36.9)	2.91 (1.52, 5.55)	0.001
Waist-to-hip ratio							
<0.90 cm	503	84 (16.7)	1.0		8 (9.8)	1.0	
≥0.90 cm	872	335 (38.4)	3.11 (2.37, 4.08)	0.000	91 (27.6)	3.52 (1.63, 7.59)	0.001
Waist circumference – cm							
Waist < 94	925	234 (25.3)	1.0		40 (17.4)	1.0	
Waist ≥ 94	450	185 (44.1)	2.06 (1.62, 2.62)	0.000	59 (32.4)	2.28 (1.44, 3.61)	0.000
Smoking ever							
Never	827	254 (30.7)	1.0		50 (20.0)	1.0	
Ever	548	165 (30.1)	0.97 (0.76, 1.22)	0.81	49 (30.2)	1.73 (1.09, 2.73)	0.081
Smokeless tobacco							
	1162	346 (29.8)	1.0		83 (24.5)	1.0	
Ever	150	53 (35.3)	1.29 (0.90, 1.84)	0.17	12 (23.1)	0.92 (0.46, 1.85)	0.826
Drinking alcohol							
Never	1119	302 (26.9)	1.0		82 (27.4)	1.0	
Ever	130	45 (34.6)	1.43 (0.97, 2.10)	0.07	10 (22.2)	0.75 (0.35, 1.59)	0.670
Walk for at least 30 minutes daily							
Yes	1018	267 (26.2)	1.0		65 (25.0)	1.0	
No	314	134 (42.7)	2.09 (1.61, 2.72)	0.002	33 (24.8)	0.99 (0.61, 1.60)	0.463
Family history of hypertension							
No	887	223 (25.1)	1.0		52 (23.5)	1.0	
Yes	249	91 (36.5)	1.71 (1.27, 2.31)	0.000	38 (43.2)	2.47 (1.46, 4.17)	0.001
Known diabetic							
No	1261	358 (28.4)	1.0		66 (18.7)	1.0	
Yes	114	61 (53.5)	2.90 (1.97, 4.28)	0.000	33 (55.0)	5.29 (2.98, 9.41)	0.000

**Table 3:** Adjusted odds ratios (AOR) of factors correlated with hypertension prevalence, awareness, in South Asian Immigrants Al Ain, Abu Dhabi, UAE, 2012 (n = 1,375)

	Hypertension		Awareness	
Characteristics	AOR (95 % CI)	p	AOR (95 % CI)	p
Nationality				
India	1.0		1.0	
Pakistan	1.17 (0.74, 1.86)	0.496	2.40 (0.89, 6.49)	0.084
Bangladesh	0.98 (0.64, 1.50)	0.930	1.65 (0.62, 4.31)	0.310
Age group				
18-35 years	1.0		1.0	
36-45 years	1.82 (1.17, 2.85)	0.008	1.25 (0.43, 3.61)	0.675
46+ years	4.76 (2.78, 8.13)	<0.001	4.31 (1.32, 14.05)	0.015
Monthly income – AED	1.00 (0.99, 1.01	0.873		
Education				
No formal schooling	0.65 (0.26, 1.59)	0.593	0.52 (0.06, 4.55)	0.554
Primary or middle	1.22 (0.58, 2.56)	0.825	1.52 (0.32, 7.30)	0.596
Secondary or high school	1.27 (0.64, 2.49)	0.482	1.82 (0.43, 7.57)	0.408
College or university	1.0		1.0	
Occupation				
Driver	0.84 (0.49, 1.44)	0.670	2.54 (0.62, 10.45)	0.197
Laborer	1.03 (0.57, 1.88)	0.918	1.0	
Construction worker	1.19 (0.60, 2.37)	0.616	1.46 (0.31, 6.96)	0.634
Agriculture worker	1.0		0.81 (0.17, 3.81)	0.790
Salesman	1.89 (0.88, 4.04)	0.099	3.01 (0.64, 14.16)	0.162
Professional, office worker	1.28 (0.55, 3.01)	0.562	0.94 (0.14, 6.21)	0.952
Business/shop keeper	1.02 (0.40, 2.57)	0.968	1.21 (0.20, 7.29)	0.831
Hospitality worker	0.88 (0.38, 2.04)	0.766	5.99 (0.81, 44.08)	0.079
Tailor	0.55 (0.19, 1.64)	0.286	0.61 (0.04, 8.77)	0.717
Other	0.94 (0.33, 2.64)	0.903	2.11 (0.22, 19.78)	0.513
Type of accommodation				
Shared with non-relatives	1.22 (0.71, 2.11)	0.469	2.54 (0.62, 10.45)	0.197
Shared with family	0.92 (0.43, 1.96)	0.837	1.19 (0.21, 6.68)	0.846
Single accommodation	1.32 (0.66, 2.64)	0.433	1.78 (0.36, 8.86)	0.477
Live with sponsor	1.16 (0.59, 2.27)	0.670	0.77 (0.14, 4.02)	0.754
Live in a labor camp	1.0		1.0	
Length of stay in UAE				
1 to 5 years	1.0		1.0	
6 to 10 years	0.97 (0.61, 1.53)	0.890	5.47 (1.65, 18.23)	0.006
>10 years	1.04 (0.65, 1.67)	0.865	4.35 (1.32, 14.39)	0.016
Body mass index – kg/m [2]				
<25.0	1.0		1.0	
25.0-29.9	1.51 (1.04, 2.19)	0.030	1.22 (0.53, 2.84)	0.629
≥30.0	2.39 (1.38, 4.16)	0.002	1.23 (0.42, 3.62)	0.703
Waist-to-hip ratio				
<0.90 cm	1.0		1.0	
≥0.90 cm	1.78 (1.18, 2.69)	0.006	3.01 (0.96, 9.64)	0.058
Smoking ever				
Never	1.0			
Ever	0.90 (0.64, 1.27)	0.560	0.94 (0.46, 1.94)	0.880
Drinking alcohol				
Never	1.0		1.0	
Ever	1.49 (0.89, 2.48)	0.123	0.90 (0.32, 2.54)	0.847
Walk for at least 30 minutes daily				
Yes	1.0		1.0	
No	1.72 (1.15, 2.58)	0.008	1.22 (0.97, 5.08)	0.059
Family history of hypertension				
No	1.0		1.0	
Yes	1.56 (1.05, 2.32)	0.029	4.91 (2.09, 11.55)	<0.001
Known diabetic				
No	1.0		1.0	
Yes	1.23 (0.72, 2.08)	0.443	5.76 (2.21, 15.00)	<0.001

Table 3 shows the factors correlated with prevalence and awareness of hypertension, after adjusting for the potential confounders including age, income, education, occupation, residency, cigarette smoking and alcohol use. Independent risk factors for hypertension included increase in age over 35 years, overweight and obesity, central obesity, lower levels of physical activity, and family history of hypertension. However, the difference in prevalence of hypertension was not statistically significant across occupational categories. We did not find a statistically significant increase in the prevalence of hypertension among immigrants as the duration of their stay increased. We noted that the awareness increased with increase in the duration of residency for more than five years in the UAE, having a family history of hypertension, and if the participant had been diagnosed with diabetes (Table 3).

## Discussion

The prevalence of hypertension in our sample of young South Asian adult immigrant males was higher than published estimates in home countries: India (34.6 % versus 22.8 %) [22], Pakistan (28.2 % versus 18.0 %) [23] and Bangladesh (28.8 % versus 11.3 %) [24]. The overall prevalence of hypertension in the study population (30.5 %) was also higher than the native Emirati population (14.0 %) and in Arab immigrants (21.9 %) during same the study year [25]. The prevalence of hypertension among study participants aged 46 years and older was particularly high (55.5 %) compared to a recent study that reported sex-specific and age-specific prevalence of hypertension in representative population samples from all over the world [26]. In this study Kearney and colleagues reported lower age-specific prevalence rates of hypertension for men aged 40–49 years and 50–59 years from India (24.8 % and 32.6 %), Egypt and Turkey (26.1 % and 37.2 %), Sub Saharan Africa (38.5 % and 48.1 %), and an aggregated rate for USA, Canada and Japan (32.6 % and 44.8 %) grouped as established market economies. Our study findings fill an important gap in the literature pertaining to migrant worker health; namely, local estimates of chronic disease risk factors (viz. hypertension) among immigrant populations residing in the UAE. Prevalence statistics are vital for researchers, practitioners, and policy makers that are interested in surveillance, screening and developing cost-effective interventions for this at-risk population.

Our findings are in agreement with other studies [27] reporting that among the three nationalities Indians had the highest mean systolic and diastolic blood pressure and prevalence rates of hypertension, followed by Pakistanis and then Bangladeshis. In the present study, both overall obesity (indexed by BMI) and central obesity were strongly associated with an increased prevalence of hypertension. After adjustment of confounding variables, the study showed a significant relationship between hypertension and advancing age, lower levels of physical activity, obesity, central obesity, and family history of hypertension. These findings are in agreement with several other studies [28–30]. Alarmingly, 76 % of participants classified as hypertensive were not aware of their condition and 62 % of study participants had never had their blood pressure examined. The lack of awareness in the study population compares with home-country populations in India (46.7 %) [31], Pakistan (70.0 %) [23] and Bangladesh (62.0 %) [32]. A clinic-based study in Al Ain showed that under-diagnosis of hypertension among non-Emiratis was much more common than UAE nationals [33]. In our study treatment and control of hypertension was very low. This underscores the urgent need for strategies to reduce inadequate control of hypertension. A high proportion (68 %) of participants had a rural background and studies have shown that people with rural background and low socioeconomic status have limited access to medical care resulting in poor control of blood pressure [34, 35].

Our findings suggest that the assessment of common risk factors for NCDs such as body mass and blood pressure measurement should be considered for inclusion in the battery of tests required as part of the medical examination at the time of applying or renewing visas for immigrants in the UAE. In this study, there was a high prevalence of hypertension awareness among those who had a family history of hypertension, those who were diabetic, and those who had lived in UAE for five or more. Previous research has shown that familial history of high blood pressure in a first-degree relative increases the likelihood by about twofold that an individual will have high blood pressure [36]. The high rate of awareness among diabetics is probably because they receive more frequent contact with primary health care providers [37]. Although a basic health insurance system has been developed for the working population in the UAE, a significant proportion of the study population, particularly those who have been in UAE for less than five years remained unaware of their hypertension status. In this study, of those aware of their hypertension, less than half reported use of antihypertensive drugs in the past two days and of these only 8.3 % had their hypertension controlled to 140/90 mm Hg or below. Treatment and control of hypertension is crucial for reducing cardiovascular death rates as the study population is already prone to developing coronary heart disease at a younger age (<40 in men) [38] and have a 3- to 5-fold increase in the risk for myocardial infarction and cardiovascular death compared with other ethnic groups [39, 40]. As such, there should be more emphasis on improving the provision of primary health care services to South Asians living in the UAE coupled with educational campaigns to encourage people to check their BP more regularly, particularly if they have familial history of hypertension and/or diabetes.

### Limitations of the study

There a number of study limitations that needs to be acknowledged. Firstly, the cross sectional design does not permit a causal relationship between risk factors and hypertension. Secondly, we recruited the sample in the only visa screening centre in the city of Al Ain (Abu Dhabi Emirate). However, we would not expect the socioeconomic and lifestyle characteristics of the study population to differ from South Asian male immigrants living in other Emirates of the UAE. Due to funding limitation we did not measure lipid levels and only measured HbA1c levels in a small sub-sample (n = 99) of study population. We have included current medication as taking medicine in the last two days instead of daily. Other studies have similarly included those taking medications less than daily [41]. Nonetheless; this is the first population-based study to report the prevalence of hypertension among a representative sample of South Asian male immigrants living in the UAE.

## Conclusions

Our study revealed a high prevalence of hypertension among a relatively young South Asian male immigrant population living in the UAE. Based on the findings of our study, we would like to recommend that blood pressure, height and body mass measurements should be included in the medical screening tests at the time of obtaining or renewing a residency visa for expatriates living in the UAE. Population-based public health interventions targeting the maintenance of a healthy body size (both total and central adiposity), coupled with regular assessment of blood pressure in South Asian male immigrants are urgently required. Future initiatives need to consider the sociocultural, religious, ethnic, and educational diversity of this population in the design, development, and implementation of campaigns, interventions, and strategies.

## Abbreviations

AED: Emirati Dirham
AOR: Adjusted Odds Ratio
BMI: Body Mass Index
BP: Blood Pressure
CI: Confidence Interval
DBP: Diastolic Blood Pressure
DPSC: Disease Prevention and Screening Centre
HbA1c: Glycated haemoglobin
IPAQ: International Physical Activity Questionnaire
NCD: Non-communicable Chronic Diseases
SBP: Systolic Blood Pressure
UAE: United Arab Emirates
USA: United States of America
USD: United States Dollar
WHO: World Health Organization
WC: Waist circumference
WHR: Waist-to-Hip Ratio
